# The relationship between famine exposure during early life and body mass index in adulthood: A systematic review and meta-analysis

**DOI:** 10.1371/journal.pone.0192212

**Published:** 2018-02-06

**Authors:** Jielin Zhou, Liangjian Zhang, Peng Xuan, Yong Fan, Linsheng Yang, Chunqiu Hu, Qingli Bo, Guoxiu Wang, Jie Sheng, Sufang Wang

**Affiliations:** School of Public Health, Anhui Medical University, Hefei, Anhui, P. R. China; CUNY, UNITED STATES

## Abstract

**Background:**

Previous epidemiologic studies have reported famine exposure during early life association with overweight or obesity in adulthood, but a consistent perspective has not been established to date.

**Purpose:**

To determine, by conducting a systematic review and meta-analysis, whether exposure to famine could increase body mass index (BMI) in adult or not, and assess the association between famine exposure and the risk of overweight or obesity.

**Methods:**

Published articles were systematically searched (until August, 2017) from PubMed, ScienceDirect, Cochrane, and China National Knowledge Infrastructure. Initially, comparing differences in BMI between exposed and non-exposed groups that weight mean difference (WMD) were used. Subsequently, the effect of famine exposure on overweight or obesity risk, which pooled relative risks (RRs), odds ratios (ORs) or hazard ratios (HRs) with 95% confidence intervals (CIs) were calculated using a random-effects model.

**Result:**

Twenty studies were included in this systematic review and meta-analysis. Compared with non-exposed group, famine exposure group significantly increased the risk of overweight (OR = 1.10, 95% CI: 1.04–1.16) and obesity (OR = 1.15, 95% CI: 1.05–1.24). Sensitivity analyses revealed no significant change in the famine exposure and BMI, the risk of overweight and obesity study when any one study was excluded. Subgroup analyses showed that age, gender, exposure type, study type, continent, famine cause and paper publication date were associated with BMI, the risk of overweight and obesity. Meta-regression analyses suggested that continent, famine cause could partially explain heterogeneity for famine exposure and BMI studies.

**Conclusion:**

The systematic review and meta-analysis indicates that famine exposure during early life may increase BMI, the risk of overweight and obesity, especially for female, fetal famine exposure or subject age less than 50. Furthermore, famine exposure group the risk of overweight and obesity in cross-sectional studies, Asian studies, famine cause by natural disaster or paper published from 2015 to the present studies are higher than that of non-exposed group.

## Introduction

Body mass index (BMI) was defined as weight in kilograms divided by the height of the square in meters [1], which is far more commonly used to define overweight or obesity and closely related to the degree of body fat in most settings. As the economy grows and living standards improves rapidly, the prevalence of overweight and obesity increases as well. In 2010, overweight and obesity approximately resulted in 3.8% of DALYs, 3.4 million deaths, and 3.9% of years of life lost globally [2]. Besides, overweight and obesity are critical risk factors for cardiovascular disease and linked status, including hypercholesterolemia, hypertension, type 2 diabetes, coronary heart disease, and stroke [3]. Study by Lim SS et al. [4] have suggested that unabated the rise in obesity could well response for future declines in life expectancy.

Previous research had reported that, several risk factors were confirmed to be significantly associated with overweight and obesity [5], for instance, cigarette smoking, alcohol consumption, high fat dietary, physical inactivity behavior, genetic and environmental factors, various chronic diseases factors (hypertension, diabetes, dyslipidemia and so on) [6]. Recently, presenting a branch of scientific knowledge, known as the developmental origins of health and disease (DOHaD) [7], covering its concepts that human in the early stages of the development process (including the fetus, infant, childhood) experience adverse factors (uterine placental dysfunction, malnutrition, etc.) would affect adult the occurrence of obesity, diabetes, cardiovascular disease and so on. Furthermore, famine exposure during early life may alter neuroendocrine function and induce HPA axis to release excessive glucocorticoid, which would increase BMI, the risk of overweight and obesity [8]. Compelling evidence have been performed to explore the relationship between exposure to famine during early life and the risk of overweight and obesity in adulthood. However, the results are controversial. Study of GP Ravelli et al. [9] found that early life was stimulated by Dutch famine could increase obesity prevalence in adult. In addition, the majority of research had reported that the Chinese great famine brought about shorter stature and overweight in females after 50 years [10–11]. Whereas several studies indicated no significant association between famine exposure during early life and adult overweight and obesity risk, such as Li yuanbi et al and Zhao yan et al [12–13]. Therefore, we systematically conducted a systematic review and meta-analysis to explore the relation between famine exposure during early life stage and BMI in adult, and further estimate the associations between famine exposure and the risk of overweight or obesity in adulthood.

## Methods

We carried out a systematic review and meta-analysis according to the Cochrane methodology and the recommendations for reporting proposed by the systematic review and meta-analysis of observational studies in epidemiology group [14] (S1 Table).

### Famine definition

The criteria for the definition of famine were not consistent in each region [12], such as Chinese famine was defined as food supplement dropped by 70% with the time reported from September 30, 1959 to October 1, 1961. However, the 1944–1945 Dutch famine was defined as energy supplement less than 1000kcal every day.

### Search strategy

**Fig 1:**
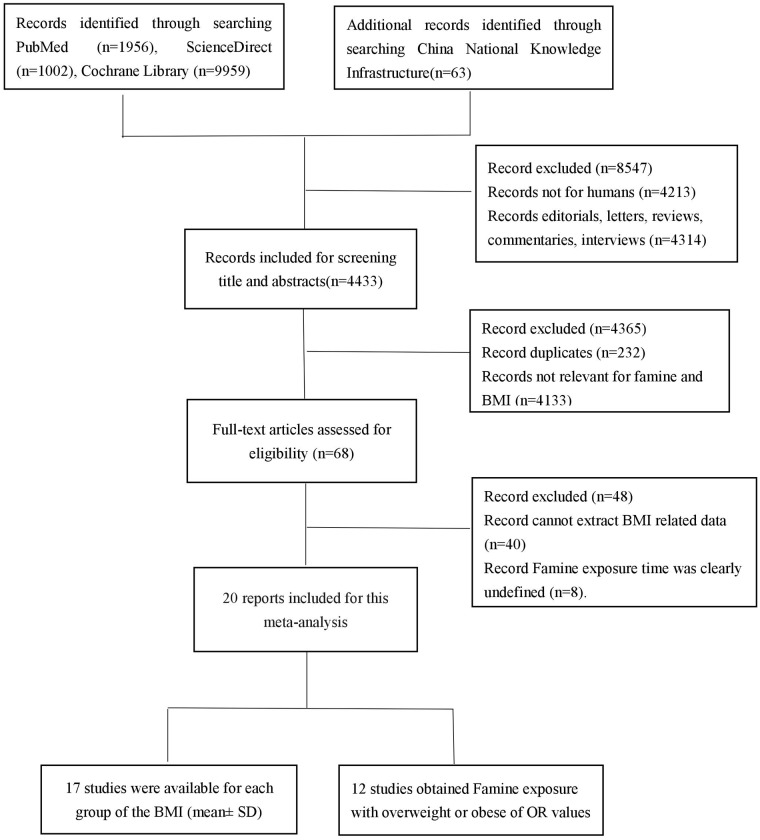
Study selection flowchart.

We conducted a literature search to identify relevant available articles with English or Chinese from PubMed, ScienceDirect, Cochrane library, and China National Knowledge Infrastructure, which published up to August 2017. The search terms, including “famine” “starvation” “hunger” “undernutrition” “undernourishment” “malnutrition” “malnourishment”, “body mass index” “BMI”, after screening titles and abstracts, two reviewers independently examined full text articles and extracted data on study characteristics, quality and results. We also reviewed the reference lists from the included articles to search for further relevant studies. The flowchart of literature search was showed in Fig 1.

### Inclusion and exclusion criteria

The inclusion criteria were as follows: (1) original article was an observational study. (2) the exposure of interest was famine. (3) BMI value (mean± SD, Standard Deviation), overweight or obesity relative risks (RRs), odds ratios (ORs) or hazard ratios (HRs) with 95% confidence intervals (CIs) were available, or calculated them by providing data. (4) the latest and most complete study was selected if data from the same participant had been published more than once. Articles were excluded based on the following criteria: (1) animal experiment rather than human study. (2) editorials, letters, reviews, commentaries or interviews. (3) duplicate articles. (4) irrelevant for famine exposure and BMI, the risk of overweight and obesity. (5) undefined famine exposure time. All identified studies were carefully reviewed independently by two investigators to determine whether an individual study was eligible for inclusion criteria in this study.

### Data extraction

The following data were extracted: the first author’s name, study continent (region), paper publication year, study language, study design type, the average age of subjects, percentage of males (%), definition standard of obesity/overweight by using BMI, famine duration period, adjustment for covariates, famine exposure grouping, BMI value (mean± SD), overweight or obesity RRs, ORs or HRs with theirs 95% CIs.

### Quality evaluation

The Newcastle-Ottawa Quality Assessment Scale was applied for literature quality evaluation [15]. Nine questions were assessed and each satisfactory answer received 1 point, causing a maximum score of 9. Only these studies in which the majority of the questions were deemed satisfactory (i.e. with a score of 6 or higher) were considered to be of high methodological quality.

### Statistical analysis

All statistical analysis were performed by using STATA, version 12.0. Originally, BMI value, as continuous data, weight mean difference (WMD) was used to compare the BMI distinction between exposed and non-exposed group. Subsequently, pooled measure was calculated as the inverse variance weighted mean of the logarithm of OR (OR, RR, HR) with theirs 95% CIs to evaluate the strength of association between famine exposure and the risk of overweight or obesity. Generally, I^2^ was used to assess heterogeneity between studies (I^2^ values of 0%, 25%, 50%, and 75% represent no, low, moderate and high heterogeneity, respectively) [16]. Less than 50% of I^2^ was considered acceptable heterogeneity in this study. Random effect model was used to estimate the pooled effect and 95% CIs. The sensitivity analysis was performed to assess the key studies with substantial impact on the between-study heterogeneity. Subgroup analyses and meta regression analyses were carried out to further find heterogeneity source by gender, age, exposure situation, study type, continent, famine cause, paper publication date, adjusting for the confounding factors variable. Publication bias was assessed with visual inspection of the funnel plot and Egger’s test [17]. The graph were symmetrical inverted funnel-shaped or Egger's test P value more than 0.05, which indicated without bias. All reported probabilities (P values) were two-sided with a statistical significance level of 0.05.

## Results

### Characteristics of studies

**Fig 2:**
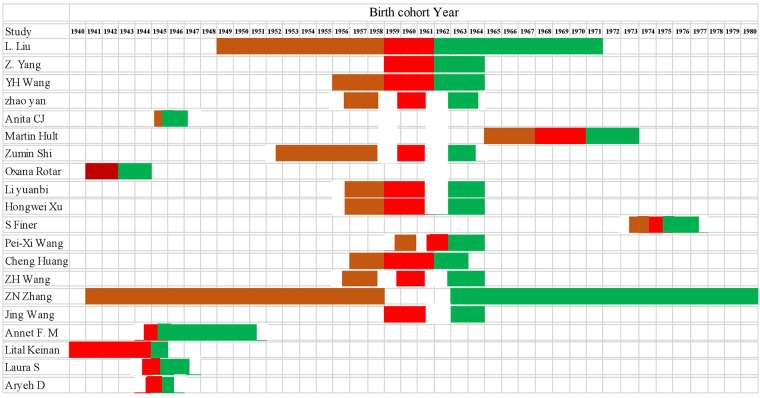
All studies grouping in the famine exposure. Red: fetal exposed, Brown: childhood exposed, Green: nonexposed.

We identified 20 published articles [10–13, 18–33], of which 17 studies with 27 data were available for each group BMI value (mean± SD), and 12 studies obtained famine exposure and the OR (OR, RR, HR) values of overweight with 27 data or obese with 42 data for this study, to estimate the relationship between famine exposure during early life and later BMI, the risk of overweight and obesity. Chinese and English study were included in this study, apart from the study of Li yuanbi et al. [12] and Zhao yan et al. [13], other 18 studies were in English. Eleven cross-sectional, six historical cohort and three prospective cohort studies were included in the present study. Among these studies, 13 studies carried out in Asia, 6 studies in Europe and 1 studies in Africa. Including 6 types famine duration period (1940–1944, 1943–1944, 1944–1945, 1959–1961, 1967–1970, 1974–1975). Fifteen studies defined overweight and obesity criteria by BMI value, of which 8 studies defined 5 kinds of overweight criteria and 8 studies defined 3 types obesity criteria. The detailed characteristics of the included studies were presented in Table 1. All studies famine exposure grouping were showed in Fig 2. Birth cohort year of the longest span was in ZN Zhang et al. [18] study, ranging from 1941 to 1980. By contrary to ZN Zhang et al. [18] study, Aryeh D. Stein et al. [19], who studied birth cohort only 15 months with the shortest time extent. In addition, Li yuanbi et al. [12] study’s birth cohort timing was consistent with Hongwei Xu et al. [20] study.

**Table 1:** Characteristics of studies for famine exposure included in this study.

Author (year)	Continent (region)	Study language	Study design	Age (mean)	Males (%)	Definition of Obesity/Overweight(kg/m^2^)	Famine duration period	Famine exposure grouping	Adjustment for covariates
YH Wang et al [10], (2009)	Asia (Chongqing)	English	Cross-sectional	NM	61.1	Overweight: 25<BMI<29.9 Obesity: BMI≥30	1959–1961	fetal, childhood exposure, nonexposure	Unadjustment
L. Liu et al [11] (2017)	Asia (Qingdao)	English	Cross-sectional	49.9	38.0	Overweight: 24<BMI<27.9 Obesity: BMI≥28	1959–1961	fetal, childhood, adolescence exposure, nonexposure	Age, sex, education, family obesity history, family month income, smoking, drinking, chronic disease history.
Li yuanbi et al [12], (2014)	Asia (Hefei)	Chinese	Cross-sectional	52.5	49.0	Obesity: BMI≥24	1959–1961	fetal, childhood exposure, nonexposure	Family year income, Feeding style, Dietary intake.
Zhao yan et al [13], (2013)	Asia (Hefei)	Chinese	Cross-sectional	NM	59.6	Overweight: 24<BMI<27.9 Obesity: BMI≥28	1959–1961	fetal, childhood exposure, nonexposure	Sex, education, smoking, family history, region, physical activities, Nutritional supplements, dietary pattern score.
ZN Zhang et al [18], (2017)	Asia (Guangdong)	English	Cross-sectional	51.7	49.1	ND	1959–1961	childhood exposure, nonexposure	Unadjustment
Aryeh D et al [19], 2009	Europe (Leiden)	English	Cross-sectional	59.0	45.3	ND	1944–1945	fetal exposure, nonexposure	Unadjustment
Hongwei Xu et al [20], (2016)	Asia (Hubei)	English	Historical cohort	NM	48.3	Overweight: BMI≥25	1959–1961	fetal, childhood exposure, nonexposure	Unadjustment
Martin Hult et al [21], (2010)	Africa (Biafran)	English	Historical cohort	39.7	61.8	Overweight: 25<BMI<29.9 Obesity: BMI≥30	1967–1970	fetal, childhood exposure, nonexposure	Unadjustment
Zumin Shi et al [22], (2013)	Asia (Jiangsu)	English	Cross-sectional	43.0	45.1	Overweight: BMI≥24	1959–1961	fetal, childhood exposure, nonexposure	Unadjustment
Oxana Rotar et al [23], (2015)	Europe (Saint Petersburg)	English	Historical cohort	70.7	27.0	Obesity: BMI≥30	1943–1944	fetal exposure, nonexposure	Unadjustment
S Finer et al [24], (2016)	Asia (Matlab)	English	Historical cohort	44.5	NM	Overweight: BMI≥23	1974–1975	fetal, childhood exposure, nonexposure	Unadjustment
Pei-Xi Wang et al [25], (2012)	Asia (Guangdong)	English	Historical cohort	49.5	49.0	Overweight: 24<BMI<27.9 Obesity: BMI≥28	1959–1961	fetal, childhood exposure, nonexposure	Age, education, occupation, smoking, drinking, physical activities, dietary habits, residence, hypertension history.
Cheng Huang et al [26], (2010)	Asia(Hebei, Zhejiang, Jiangsu)	English	Cross-sectional	31.7	0.0	Overweight: BMI≥25	1959–1961	fetal, childhood exposure, nonexposure	Unadjustment
ZH Wang et al [27], (2016)	Asia (Nationwide)	English	Cross-sectional	NM	49.3	Overweight: BMI≥24	1959–1961	fetal, childhood exposure, nonexposure	Unadjustment
Jing Wang et al [28], (2017)	Asia (Hubei)	English	Prospective cohort	56.4	16.8	Overweight: BMI≥24	1959–1962	fetal, childhood exposure, nonexposure	Unadjustment
Lital Keinan et al [29], (2015)	Europe (Jewish)	English	Cross-sectional	69.3	49.6	ND	1940–1944	fetal exposure, nonexposure	Unadjustment
LauraS et al [30], 2016	Europe (Amsterdam)	English	Prospective cohort	68.0	45.0	ND	1944–1945	fetal exposure, nonexposure	Unadjustment
Annet F. M et al [31], (2013)	Europe (Arnhem)	English	Prospective cohort	59.8	0.0	ND	1944–1945	fetal exposure, nonexposure	Unadjustment
Anita CJ et al [32], (1999)	Europe (Amsterdam)	English	Cross-sectional	50.0	48.0	Overweight:BMI≥25	1944–1945	childhood exposure, nonexposure	Unadjustment
Z.Yang et al [33], (2008)	Asia (Nationwide)	English	Historical cohort	NM	45.6	Overweight: 24<BMI<27.9 Obesity: BMI≥28	1959–1961	fetal exposure, nonexposure	Geographic areas

NM: Not mentioned, ND: No definition

### Quality assessment

The results of the literature quality evaluation were shown in S2 Table. There were 18 articles in high methodological quality, including YH Wang et al. [10], L. Liu et al. [11], Li yuanbi et al. [12], Zhao yan et al. [13], ZN Zhang et al. [18], Hongwei Xu et al. [20], Martin Hult et al. [21], Zumin Shi et al. [22], Oxana Rotar et al. [23], S Finer et al. [24], Pei-Xi Wang et al. [25], Cheng Huang et al. [26], ZH Wang et al. [27], Jing Wang et al. [28], Lital Keinan et al. [29], Laura S. et al. [30], Anita CJ et al. [32] and Z. Yang et al. [33] research. Besides, Aryeh D. et al. [19] and Annet F. M et al. [31] study had low methodological quality.

### Quantitative synthesis

**Fig 3:**
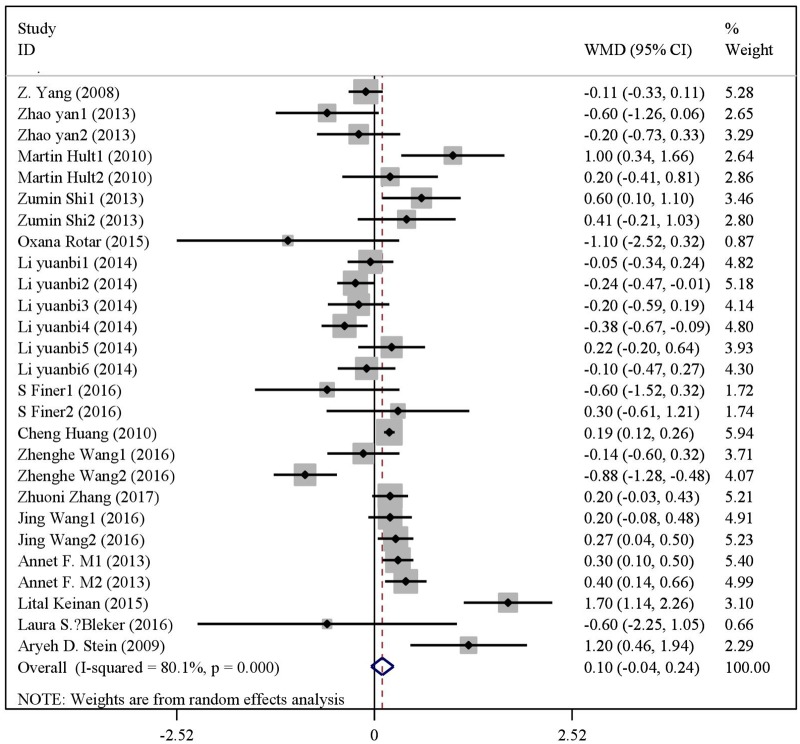
The forest plot of famine exposure and BMI.

**Fig 4:**
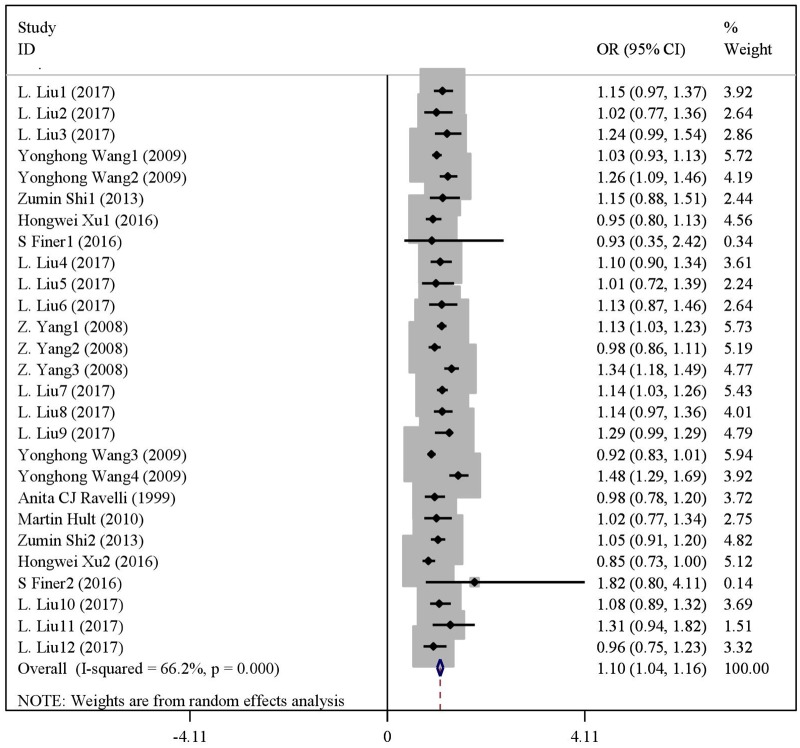
The forest plot of famine exposure and the risk of overweight.

**Fig 5:**
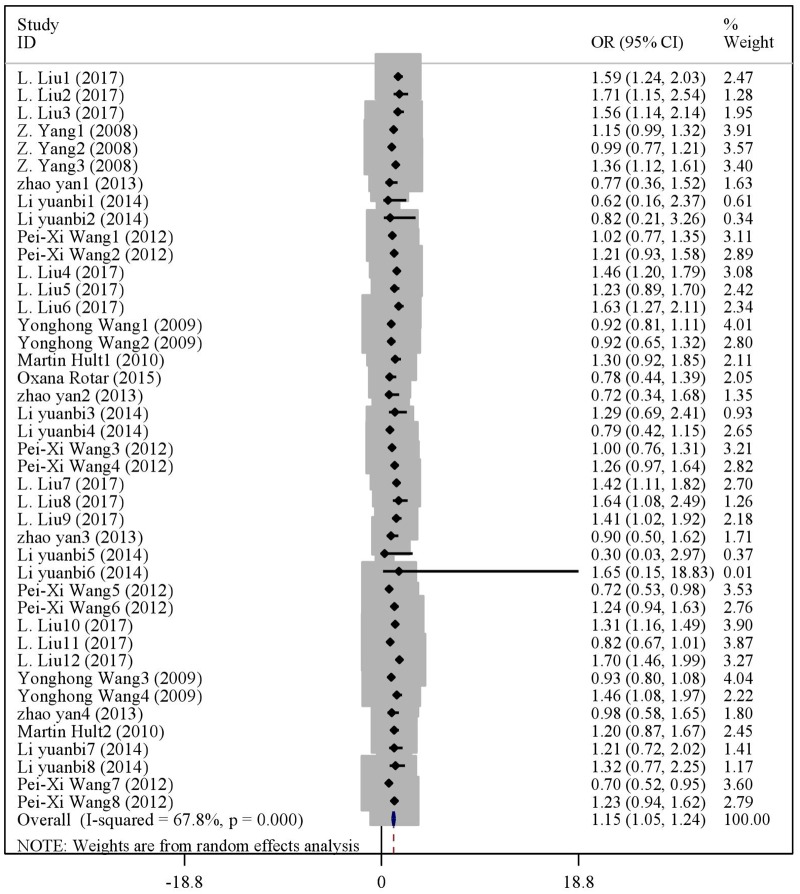
The forest plot of famine exposure and the risk of obesity.

There were statistical distinction between famine exposed group and nonexposed group in BMI (WMD = 0.10, 95% CI: -0.04–0.24) (Fig 3). But significant difference was observed in the risk of overweight (OR = 1.10, 95% CI: 1.04–1.16) (Fig 4) and obesity (OR = 1.15, 95% CI: 1.05–1.24) (Fig 5). The results of subgroup analysis were presented in Table 2. In age subgroup analysis, there were higher BMI level (WMD = 0.33, 95% CI: 0.06–0.60) and risk of overweight (OR = 1.13, 95% CI: 1.07–1.19) or obesity (OR = 1.24, 95% CI: 1.08–1.39) in age less than 50. In gender subgroup analysis, famine exposure during early life could significantly increase BMI (WMD = 0.22, 95% CI: 0.11–0.33), the risk of overweight (OR = 1.26, 95% CI: 1.15–1.37) and obesity (OR = 1.30, 95% CI: 1.16–1.45) in female. By contrast, the result was not observed in male. In exposure type subgroup analysis, fetal exposure could increase BMI (WMD = 0.22, 95% CI: 0.05,0.38), the risk of overweight (OR = 1.11, 95% CI: 1.04–1.18) or obesity (OR = 1.15, 95% CI: 1.04–1.26) in adulthood, but the result was not found in childhood exposure. In study type subgroup, we found that cross-sectional studies, famine exposure group risk of overweight (OR = 1.12, 95% CI: 1.05–1.19) and obesity (OR = 1.20, 95% CI: 1.07–1.34) were higher than non-exposed group. In continent subgroup, we found that Asian studies, famine exposure group risk of overweight (OR = 1.11, 95% CI: 1.04–1.17) and obesity (OR = 1.15, 95% CI: 1.05–1.25) were higher than non-exposed group. In famine cause subgroup analysis, we found that natural disaster, famine exposure risk of overweight (OR = 1.11, 95% CI: 1.04–1.17) and obesity (OR = 1.15, 95% CI: 1.05–1.25) were higher than non-exposed group. In paper publication date subgroup analysis, we found that the paper published from 2015 to the present, famine exposure risk of overweight (OR = 1.09, 95% CI: 1.01–1.16) and obesity (OR = 1.30, 95% CI: 1.13–1.47) were higher than non-exposed group.

**Table 2:** The subgroup analysis between famine exposure and BMI, the risk of overweight, obesity.

Type	Subgroup	Studies numbers	WMD (95 CI%)	Heterogeneity			Publication bias	
				χ^2^	I^2^ (%)	P	T	P
Famine exposure and BMI	All studies	27	0.10(-0.04,0.24)	130.47	80.1	<0.001	-0.65	0.522
	Gender							
	Male	3	-0.21(-0.37,-0.04)	2.10	4.8	0.350	-1.81	0.670
	Female	5	0.22(0.11,0.33)	5.82	31.3	0.213	0.23	0.834
	Male/Female	19	0.14(-0.10,0.38)	99.40	81.9	<0.001	0.33	0.777
	Age							
	NM	5	-0.36(-0.69,-0.04)	12.22	67.3	0.016	-1.98	0.398
	≤50	7	0.33(0.06,0.60)	11.62	48.4	0.071	0.58	0.425
	>50	15	0.17(-0.03,0.38)	77.59	82.0	<0.001	0.38	0.808
	Exposure type							
	Fetal	18	0.22(0.05,0.38)	75.02	77.3	<0.001	0.07	0.925
	Childhood	9	-0.11(-0.38,0.15)	33.88	76.4	<0.001	0.11	0.954
	Study type							
	Historical cohort	6	0.07(-0.40,0.53)	14.65	65.9	0.012	0.35	0.801
	Cross sectional	16	0.06(-0.14,0.27)	104.04	85.6	<0.001	-0.64	0.523
	Prospective cohort	5	0.29(0.17,0.41)	2.23	0.0	0.694	-1.15	0.197
	Continent							
	Asia	19	-0.04(-0.18,0.10)	73.44	75.5	<0.001	-1.46	0.307
	Africa	2	0.59(-0.19,1.37)	3.06	67.3	0.08	-	-
	Europe	6	0.56(0.06,1.07)	31.36	84.1	<0.001	0.61	0.761
	Famine cause							
	Natural disaster	17	-0.03(-0.18,0.11)	71.06	77.5	<0.001	-1.62	0.043
	War	10	0.46(0.09,0.83)	39.72	77.3	<0.001	0.06	0.961
	Publication date							
	Before 2010	5	0.34(0.02,0.66)	20.12	80.1	<0.001	1.09	0.509
	2011 to 2014	12	0.02(-0.18,0.21)	40.94	73.1	<0.001	-0.74	0.666
	2015 to present	10	0.05(-0.33,0.43)	63.6	85.8	<0.001	-0.95	0.585
Famine exposure and overweight	All studies	27	1.10(1.04,1.16)	76.84	66.2	<0.001	0.21	0.832
	Exposure type							
	Fetal	14	1.11(1.04,1.18)	23.25	44.1	0.039	-0.27	0.763
	Childhood	13	1.09(0.99,1.20)	51.26	76.6	<0.001	0.76	0.623
	Confounding							
	Unadjusted	18	1.10(1.12,1.18)	59.17	71.3	<0.001	0.64	0.571
	Adjusted	9	1.11(1.02,1.20)	15.13	47.1	0.057	-0.71	0.548
	Gender							
	Male	7	1.00(0.93,1.07)	7.71	22.2	0.26	1.43	0.18
	Female	7	1.26(1.15,1.37)	12.12	50.5	0.059	-3.26	0.054
	Male/Female	13	1.15(0.99,1.12)	17.79	32.6	0.122	-0.32	0.666
	Age							
	NM	9	1.05(0.98,1.21)	59.07	86.5	<0.001	0.75	0.872
	≤50	13	1.13(1.07,1.19)	9.63	0	0.648	-0.18	0.764
	>50	5	1.07(0.95,1.19)	2.52	0	0.642	-0.09	0.961
	Study type							
	Cross sectional	19	1.12(1.05,1.19)	47.92	62.4	<0.001	0.76	0.475
	Historical cohort	8	1.05(0.92,1.18)	28.21	75.2	<0.001	-0.63	0.691
	Continent							
	Asia	25	1.11(1.04,1.17)	75.94	68.4	<0.001	0.39	0.702
	Africa	1	1.02(0.73,1.31)	-	-	-	-	-
	Europe	1	0.98(0.77,1.20)	-	-	-	-	-
	Famine cause							
	Natural disaster	23	1.11(1.04,1.17)	75.1	70.7	<0.001	0.27	0.808
	War	4	1.00(0.84,1.17)	0.99	0	0.803	0.85	0.402
	Publication date							
	Before 2010	9	1.12(1.01,1.23)	48.61	83.5	<0.001	1.15	0.709
	2011 to 2014	2	1.07(0.94,1.21)	0.31	0	0.576	-	-
	2015 to present	16	1.09(1.01,1.16)	27.82	46.1	0.023	-0.06	0.939
Famine exposure and obesity	All studies	42	1.15(1.05,1.24)	127.5	67.8	<0.001	-0.94	0.353
	Exposure type							
	Fetal	23	1.15(1.04,1.26)	47.79	54	0.001	-0.46	0.523
	Childhood	19	1.15(0.99,1.31)	78.37	77	<0.001	-0.63	0.526
	Confounding							
	Unadjusted	23	1.12(1.00,1.25)	84.59	74	<0.001	-0.72	0.457
	Adjusted	19	1.18(1.04,1.32)	40.15	55.2	0.002	-0.48	0.483
	Gender							
	Male	15	0.93(0.84,1.03)	23.14	39.5	0.058	0.67	0.42
	Female	15	1.30(1.16,1.45)	26.38	46.9	0.023	-1.4	0.061
	Male/Female	12	1.21(1.081.35)	18.63	41	0.068	-1.17	0.086
	Age							
	NM	11	1.04(0.92,1.15)	19.48	48.7	0.035	-0.46	0.644
	≤50	17	1.24(1.08,1.39)	68.45	76.6	<0.001	-2.67	0.07
	>50	14	1.26(0.90,1.35)	26.39	50.7	0.015	-0.15	0.856
	Study type							
	Cross sectional	28	1.20(1.07,1.34)	91.47	70.5	<0.001	-0.31	0.648
	Historical cohort	14	1.07(0.95,1.19)	34.32	62.1	0.001	-1.88	0.164
	Continent							
	Asia	39	1.15(1.05,1.25)	124.83	69.6	<0.001	-0.82	0.417
	Africa	2	1.24(0.94,1.55)	0.1	0	0.749	-	-
	Europe	1	0.78(0.31,1.26)	-	-	-	-	-
	Famine cause							
	Natural disaster	39	1.15(1.05,1.25)	124.83	69.6	<0.001	-0.5	0.417
	War	3	1.11(0.81,1.40)	2.64	24.2	0.267	-3.62	0.221
	Publication date							
	Before 2010	9	1.09(0.99,1.32)	19.56	59.1	0.012	1.53	0.389
	2011 to 2014	12	0.99(0.85,1.13)	21.48	48.8	0.029	-1.62	0.202
	2015 to present	21	1.30(1.13,1.47)	62.81	68.2	<0.001	-0.71	0.277

WMD: weight mean difference, OR: odds ratio, NM: Not mentioned, -: indicates no. The pooled effect size was estimated using random-effects model.

In order to further seek heterogeneity source, sensitivity analysis and meta-regression were performed. Sensitivity analysis revealed no significant change in the famine exposure and BMI, the risk of overweight or obesity when any one study was excluded (S1–S3 Figs). To consider the variation by study quality, sensitivity analysis was performed in high-quality studies. The result indicated no significant difference in the famine and BMI when any one high-quality study was excluded (S4 Fig). Thus, we eventually use meta-regression to find the source of heterogeneity (Table 3). In famine exposure and BMI studies, the 41.05% and 35.94% origin of heterogeneity could be explained due to the continent (P = 0.008) and famine cause (P = 0.02). But for famine exposure and the risk of overweight or obesity, the result demonstrated that no covariate conferred a significant impact on between-study heterogeneity.

**Table 3:** Meta-regression to find the results of heterogeneity.

Type	Covariates	β	SE	T	P>| T |	95%CI	Adjusted R^2^(%)
Famine and BMI	Gender	0.13	0.15	0.85	0.403	-0.18, 0.44	-1.06
	Age	0.21	0.13	1.65	0.111	-0.05,0.48	11.25
	Group	-0.32	0.22	-1.47	0.153	-0.77, -0.13	12.33
	Study design	0.091	0.18	0.51	0.615	-0.28, 0.46	-4.09
	Continent	0.34	0.12	2.88	0.008	0.096, 0.58	**41.05**
	Famine cause	0.52	0.21	2.49	0.020	0.09, 0.96	**35.94**
	Publication date	-0.15	0.15	-0.97	0.339	-0.46, 0.16	-5.29

β: Regression coefficients, SE: Standard error of regression coefficients, Adjusted R2(%): the current covariate can explain the size of heterogeneity.

The funnel plot showed no evidence of significant small-study effect for the analysis BMI, the risk of overweight or obesity between nonexposed group and famine exposure (S5–S7 Figs). In addition, Egger’s test suggested no significant publication bias as a whole (P > 0.05) (Table 2).

## Discussion

As noted above, this is the first systematic review and meta-analysis to evaluate the association of famine exposure during early life with the BMI in adulthood. Our studies showed that exposure to famine during early stage significant correlation to BMI, the risk of overweight and obesity, which were consistent with Wang Y H et al. [10] and Liu L et al. [11] studies. In subgroup analysis, our study suggested that exposure to famine during early life contributed to the increase of BMI, the risk of overweight and obesity in adulthood, which were positive association with female subjects studies, fetal exposure studies, subject age less than 50 studies, sectional-cross studies, Asian studies, famine cause by natural disaster and paper published from 2015 to the present studies. Furthermore, the meta-regression results intimated that continent and famine cause could contribute to explaining partial heterogeneity for famine exposure and BMI studies.

The mechanisms of famine exposure during early life and increased BMI in adult are still not clear now. Nevertheless, several plausible biological explanations have been reported. GE Miller et al. [34] study pointed out that, people suffered from famine stress early stage in its life that would activate the HPA axis to regulate appetite behavior. Early life formatted this nerve pathway, which would form adverse phenotypes for the future, and seek out appetite stimulating in nerve impulse way. Therefore, individuals tend to engage in health hazards behavior such as high fat diet, high-energy food and physical inactivity. As we know, energy intake and consumption simultaneously exists in the body, when the long-term energy intake are greater than the consumption, and it allows the energy to accumulate in the form of adipose in vivo, especially for long-term intake of excess saturated fatty acids, trans fatty acids and cholesterol. Enduring positive energy balance further elevates BMI value, which would lead to overweight or obesity later. Study of Heidi P et al. [35] indicated that, the early stress of life was controlled by epigenetic markers that regulated histones, post-translational modifications and tissue remodeling into macrophages. Thus, these cells possessed proinflammatory tendencies and manifested as decreased responses of cytokines for sex hormones inhibitor susceptibility. In the course of life, these proinflammatory trends were exacerbated by behavior and tended to own hormonal disorders, which was the product of exposure to early stress. In the act, life early stress caused excessive threat of vigilance, lack of interpersonal relationships, self-regulation dysfunction. Additionally, postnatal accelerated “growth” or “catch-up growth” hypothesis had also suggested that increased growth rate by a nutrient-enriched diet might bring about overweight or obesity [36].

Our study indicated that there was a significant increase in overweight and obesity risk in women subjects who experienced malnutrition during early life. But the result not observed in men subjects, which was in line with other studies [32–33]. Possible reasons were as follows: firstly, a study noted that women who were exposed to famine had a higher risk of overeating than men [37]. Thus, it may increase adult BMI and then increase the risk of overweight and obesity. Secondly, women deposit fat in the abdomen [38–39], and intraabdominal obesity is related to low testosterone concentrations in male and hyperandrogenicity in female [40]. Therefore, the potential obesity mechanisms are commonly classified into 2 regimentations [41]. The first category is hypothalamic dysfunction, and the second category is abnormal levels of fat cells. Follow this view, we think our findings suggesting that the different roles in male and female support the notion that the increased level of obesity after exposure to hunger early in pregnancy are due to functional changes in the central endocrine regulatory machinery rather than to fat cell abnormalities.

In addition, we found that fetal famine exposure during early life could significantly increase BMI, the risk of overweight or obesity in adulthood, but the result was not observed in childhood famine exposure. According to DOHaD hypothesis [7], famine exposure during early stages could change the structure and function of important tissues and organs. It is an relatively irreversible process in the fetal exposure, but reversible process in childhood exposure. Increasing evidence suggest that age and BMI form an inverted U-shaped relationship [42–43]. Thus, we study found that there were higher BMI level and risk of overweight or obesity in age less than 50. In cross-sectional studies, famine exposure groups overweight and obesity risks were higher than non-exposed groups. Possible reason as follow: firstly, the cross-sectional study was grouped after the data was collected. In other words, there were natural controls in the same period, so it was comparable. Secondly, the sample came from the same target population, and randomly selected a representative sample to describe the association between exposure and the outcome, so the research results had a stronger promotion. In Asian studies, famine exposure groups overweight and obesity risks were higher than non-exposed groups. Available explanation were numbers of study, and the number in Asian were relatively more than in European and African. And also the definition criterion of overweight and obesity in Europe and Africa are higher than in Asia [44–45]. Compared with the non-exposed group, the famine caused natural disasters in the risk of overweight or obesity was higher than famine caused by the war. We speculated that the famine caused by natural disasters was more severe than the famine caused by the war. Thus, natural disasters famine exposure in early stages was more harmful to organ and tissue. Interestingly, we found paper published from 2015 to the present that famine exposure groups overweight and obesity risks were higher than non-exposed groups. To our knowledge, there were least bias in the recent paper publication, so it was more convincing and reliable. In addition, the included study numbers may have some impact on the outcomes.

According to Béjar LM et al. [46] study, Asia and Africa diet were rich in high dietary fiber, high carbohydrate, and Europe diets mainly were rich in high-fat, so it could explain some heterogeneity source. In addition, the continent led to suffering from famine exposure time were different, which may be heterogeneity source. Famine cause contained natural disaster and war in the included study, most famine studies were caused by natural disasters. Thus, it was likely to be partial heterogeneous source.

This systematic review and meta-analysis has several strengths. Firstly, compared with original individual study, our study synthesized multiple famine types such as Chinese great famine, Dutch famine, Biafran war famine, Leningrad siege famine, Bangladeshi famine, Jewish Holocaust famine, enhancing statistical power to provide more precise and reliable results. Secondly, considering the potential differences of famine exposure in fetal period and childhood period, we further explored the effects of these two periods of famine exposure on BMI, the risk of overweight and obesity. Thirdly, due to the subsurface heterogeneity, we further explored the relationship between famine exposure and BMI, overweight or obesity by gender, study design type, continent, famine cause, paper publication date, adjustment for confounding factors subgroup. Therefore, the results were more reasonable and convincing. Furthermore, funnel plot and Egger’s test showed the publication bias was undetected, indicating that the included results may be unbiased.

However, several potential limitations in our study should be considered. Firstly, famine duration period were not consistent across all included studies, ranging from 1 to 5 years, which may influence the stability of our results. Secondly, the degree of famine exposure was not provided in original article. Thus, we could not analysis the relation between the severity of famine exposure and BMI, the risk of overweight or obesity. Thirdly, the criteria were not the same for overweight and obesity defined in the literature, which were likely to exaggerate or reduce the impact of famine exposure on overweight and obesity in individual studies. Fourthly, due to some low quality articles or little literature about famine and overweight risk, which may reduce the efficiency of research. Finally, this only is a systematic review and meta-analyses, and the underlying mechanism needs a large population data and animal testing to verification.

## Conclusion

In summary, results from this systematic review and meta-analyses show that famine exposure during early life significantly increased BMI, the risk of overweight and obesity especially for subjects being adult female, exposure type being fetal period and subject age being less than 50. Furthermore, famine exposure increase the risk of overweight and obesity, which are strongly associated with cross-sectional studies, natural disasters studies, Asian studies and paper published from 2015 to the present studies.

## Supporting information

S1 Fig sensitivity analysis of famine exposure and BMI. (PDF)

S2 Fig sensitivity analysis of famine exposure and overweight risk. (PDF)

S3 Fig sensitivity analysis of famine exposure and obesity risk. (PDF)

S4 Fig sensitivity analysis of famine exposure and BMI in high-quality studies. (PDF)

S5 Fig funnel plot of famine exposure and BMI. (PDF)

S6 Fig funnel plot of famine exposure and overweight risk. (PDF)

S7 Fig funnel plot of famine exposure and obesity risk. (PDF)

S1 Table Quality Evaluation. (PDF)

S2 Table PRISMA Checklist. PRISMA 2009 Checklist.
(PDF)

S1 Data (XLSX)
