# Supplementary figures and images for: The relationship between famine exposure during early life and body mass index in adulthood: A systematic review and meta-analysis
Source: PLoS One. 2018 Feb 6;13(2):e0192212. doi: 10.1371/journal.pone.0192212 (PMC5800668; doi:10.1371/journal.pone.0192212)

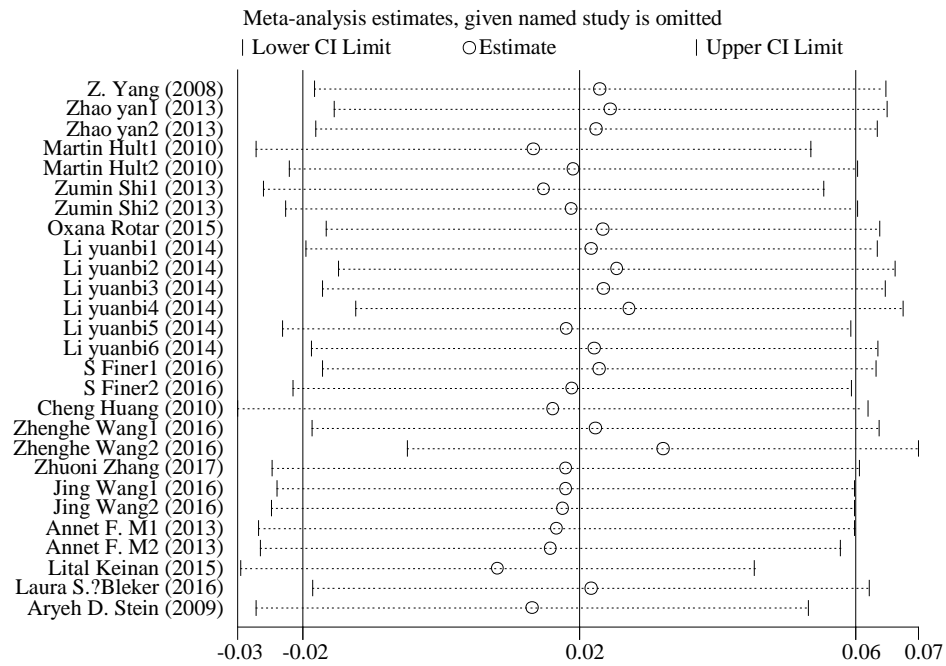

**S1 Fig. sensitivity analysis of famine exposure and BMI**

Supplement: S1 Fig — (PDF) [file pone.0192212.s001.pdf]

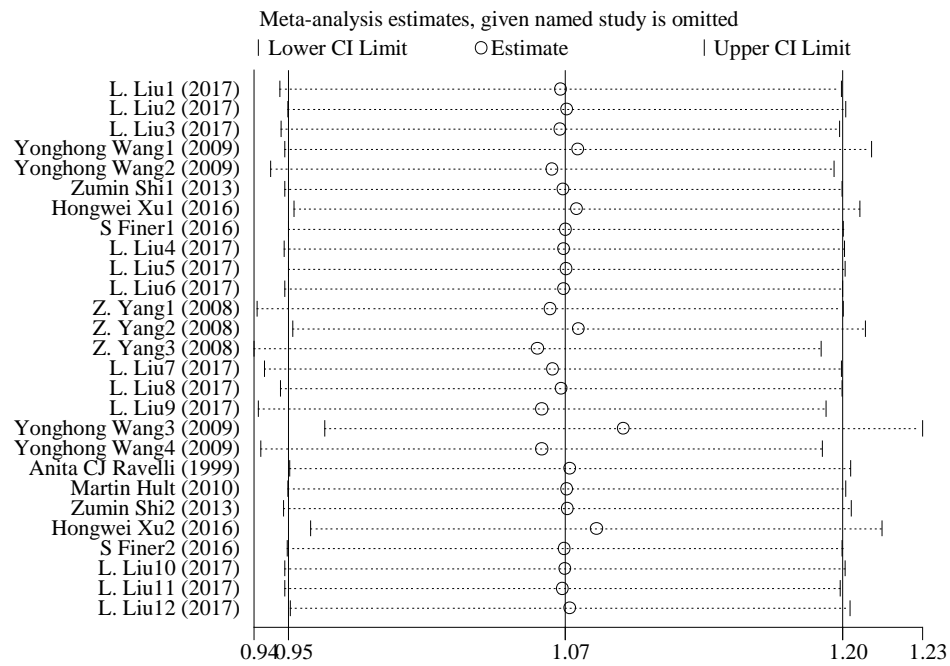

**S2 Fig. sensitivity analysis of famine exposure and overweight risk**

Supplement: S2 Fig — (PDF) [file pone.0192212.s002.pdf]

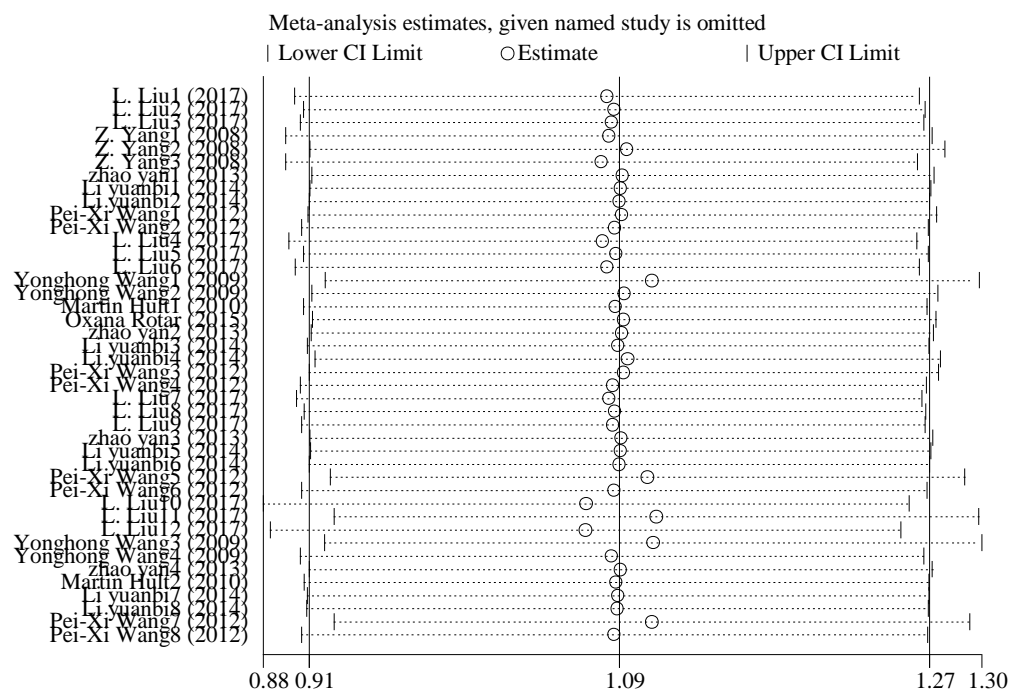

**S3 Fig. sensitivity analysis of famine exposure and obesity risk**

Supplement: S3 Fig — (PDF) [file pone.0192212.s003.pdf]

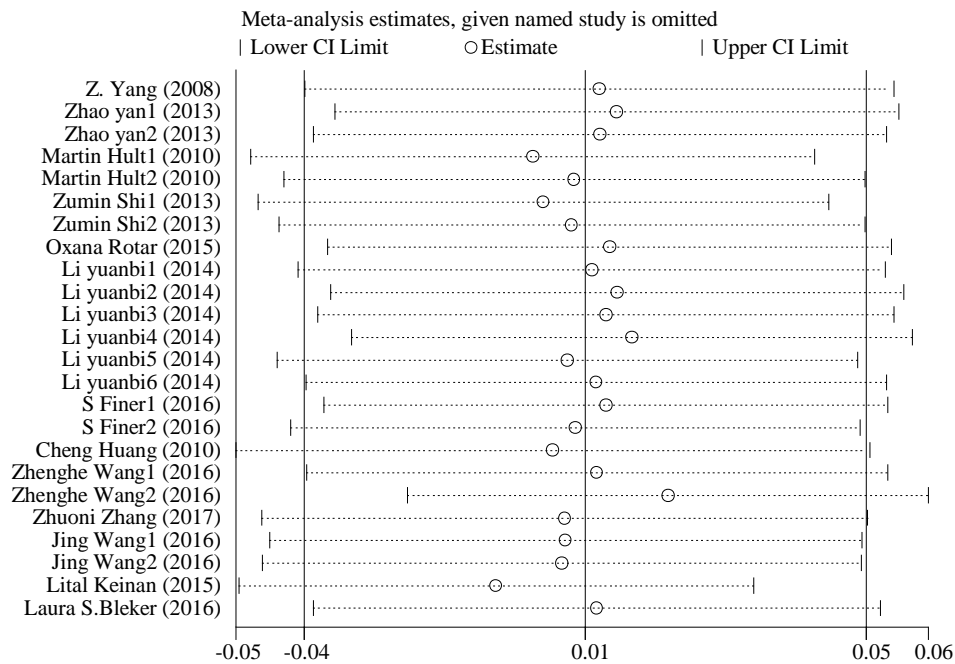

**S4 Fig. sensitivity analysis of famine exposure and BMI in high-quality studies**

Supplement: S4 Fig — (PDF) [file pone.0192212.s004.pdf]

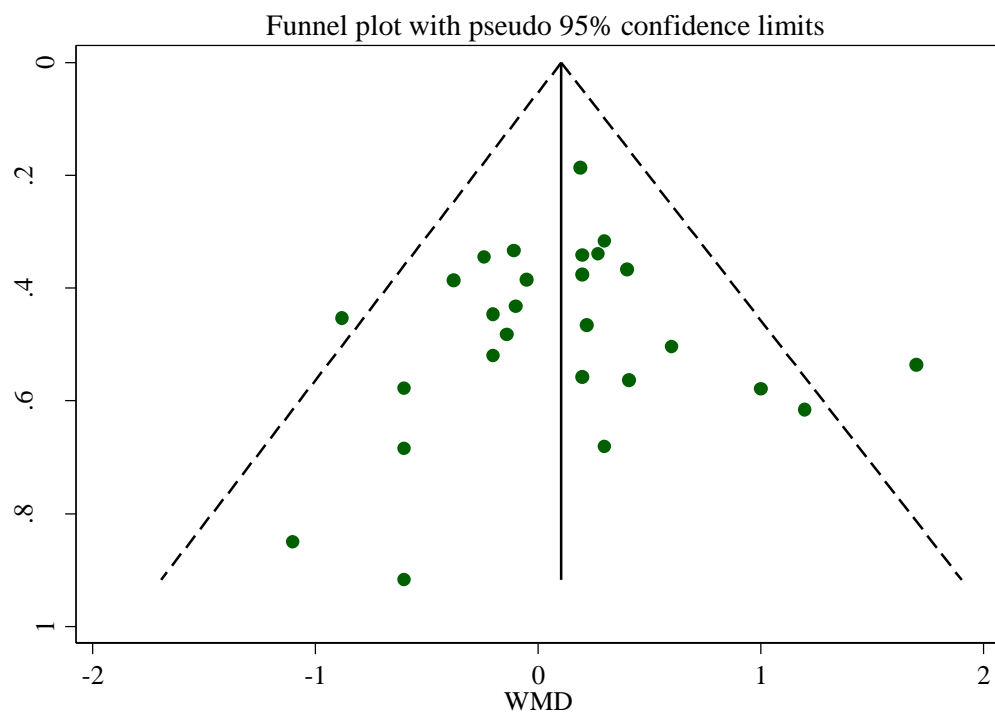

**S5 Fig. funnel plot of famine exposure and BMI**

Supplement: S5 Fig — (PDF) [file pone.0192212.s005.pdf]

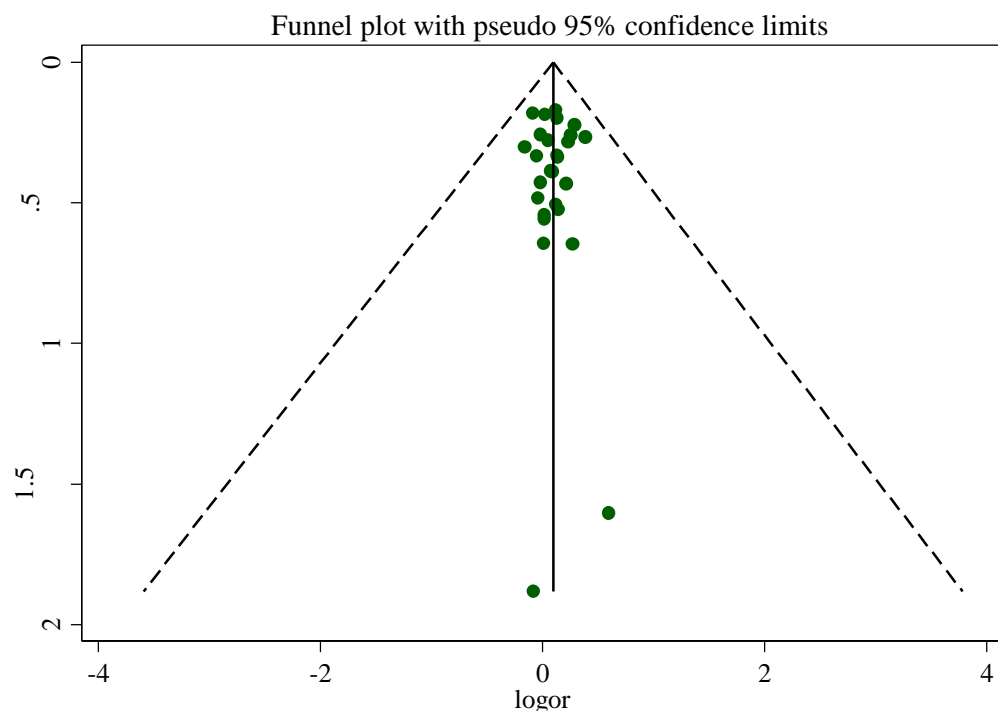

**S6 Fig. funnel plot of famine exposure and overweight risk**

Supplement: S6 Fig — (PDF) [file pone.0192212.s006.pdf]

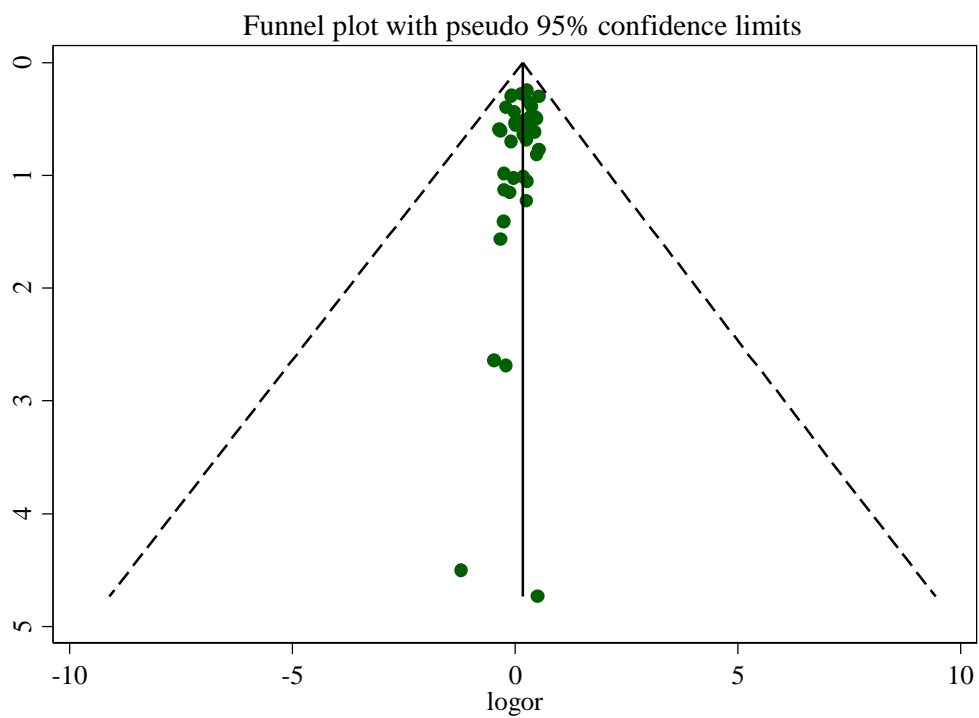

**S7 Fig. funnel plot of famine exposure and obesity risk**

Supplement: S7 Fig — (PDF) [file pone.0192212.s007.pdf]
