# Supplementary material for: The relationship between famine exposure during early life and body mass index in adulthood: A systematic review and meta-analysis
Source: PLoS One. 2018 Feb 6;13(2):e0192212. doi: 10.1371/journal.pone.0192212 (PMC5800668; doi:10.1371/journal.pone.0192212)
Supplement: S2 Table — PRISMA 2009 Checklist. (PDF) [file pone.0192212.s009.pdf]

S2 Table. Quality Evaluation

Studies	Selection				Comparability		Outcome			Score
	1	2	3	4	5A	5B	6	7	8	
YH Wang et al, 2009	*	*	*	*	*		*	*	*	********
L. Liu et al, 2017	*	*	*	*	*	*	*		*	********
Li yuanbi et al, 2014	*	*	*	*	*	*	*		*	********
Zhao yan et al, 2013	*	*	*	*	*	*	*		*	********
ZN Zhang et al, 2016	*	*	*	*	*	*	*		*	********
Aryeh D. et al, 2009	*		*	*			*		*	*****
Hongwei Xu et al, 2016	*	*	*	*	*		*		*	*******
Martin Hult et al, 2010	*	*	*	*	*	*	*		*	********
Zumin Shi et al, 2013	*	*	*	*	*	*	*	*	*	*********
Oxana Rotar et al, 2015	*	*	*	*	*	*	*	*	*	*********
S Finer et al, 2016	*	*	*	*	*		*		*	*******
Pei-Xi Wang et al, 2012	*	*	*	*	*	*	*		*	********
Cheng Huang et al, 2010	*	*	*	*	*		*	*	*	********
ZH Wang et al, 2016	*	*	*	*	*	*	*		*	********
Jing Wang et al, 2017	*	*	*	*	*	*	*	*	*	*********
Annet F. M et al, 2013	*		*	*			*		*	*****
Lital Keinan et al, 2015	*	*	*	*	*	*	*		*	********
Laura S. et al, 2016	*	*	*	*	*		*	*	*	********
Anita CJ et al, 1999	*	*	*	*	*	*	*	*	*	*********
Z. Yang et al, 2008	*	*	*	*	*		*		*	*******
